# The Impact of 12-Week Jaques-Dalcroze Eurhythmics Programme on the Dynamic Agility in Single-Dual-Task Conditions in Older Women: A Randomized Controlled Trial

**DOI:** 10.1155/2020/9080697

**Published:** 2020-07-01

**Authors:** Jan Adamczyk, Roman Celka, Rafał Stemplewski, Kinga Ceynowa, Paulina Kamińska, Janusz Maciaszek

**Affiliations:** ^1^Department of Dance and Gymnastics, Faculty of Sport Sciences, Poznań University of Physical Education, 61-871 Poznań, Poland; ^2^Department of Physical Activity Sciences and Health Promotion, Faculty of Sport Sciences, Poznań University of Physical Education, 61-871 Poznań, Poland; ^3^Faculty of Compositions, Conducting, Theory of Music and Eurhythmics, Academy of Music in Poznań, 61-808 Poznań, Poland; ^4^Graduate School for Sports Sciences, Poznań University of Physical Education, 61-871 Poznań, Poland

## Abstract

**Background:**

Progressive degenerative changes in the body of elderly people lead to a decrease in physical and mental fitness. Seniors have a problem with performing tasks that involve both physical and mental health at the same time. The risk of falls increases, the consequences of which in old age may be particularly dangerous. It was decided to investigate the impact of performing exercises involving both physical and mental spheres on the dynamic agility in older women.

**Methods:**

73 women (69.9 ± 3.2) were divided into two groups: intervention (IG, *n* = 34) and control (CG, *n* = 39). Individuals with IG participated in the Jaques-Dalcroze Eurhythmics exercise programme for 12 weeks, twice a week for 45 minutes each. Dynamic agility was determined by the Timed Up and Go test, which was conducted both in single-task (TUG_ST) and dual-task (TUG_DT) conditions, where the participant was simultaneously counting down from 60 every 3. The percentage difference between the results of both tests (dual-task cost, DTC) was also determined. Both groups had two measurement sessions: one week before the start of the exercise programme and one week after the end of exercise programme.

**Results:**

After 12 weeks of exercise, IG participants obtained significantly better results in TUG_DT (*p* < 0.001) and DTC (*p* = 0.003) tests. During this time, CG participants had significantly worse results in TUG_DT (*p* < 0.001) and DTC (*p* < 0.001) tests. In the TUG_ST test, neither IG nor CG achieved a significant change in the result. In each test, a significant interaction between the group assignment and the measurement session was observed: TUG_ST: *F* = 11.523, *η*^2^_P_ = 0.139, *p* = 0.001; TUG_DT: *F* = 60.227, *η*^2^_P_ = 0.458, *p* < 0.001; DTC: *F* = 32.382, *η*^2^_P_ = 0.313, *p* < 0.001.

**Conclusion:**

JDE exercises with a frequency of twice a week, for about 12 weeks, have a significant impact on the improvement of the dynamic agility control in women over 65 years of age.

## 1. Introduction

With age, a number of degenerative changes occur in the body of seniors. Among the most common is muscle mass loss combined with an increase in body fat [1] and osteoarthritis, which affects more than 50% of the population over 65 years of age [2]. Most cognitive functions, such as memory, logical reasoning, spatial imagination, and the speed of thought processes, are decreasing [3]. There is also a deterioration in the sensory functions, including vision responsible for maintaining body equilibrium [4]. Additionally, in old age, delirium, frailty, dizziness, and fainting are often observed—all of these disorders combine to form a geriatric syndrome and lead to a decrease in functional fitness, i.e., the ability to perform basic daily activities on their own and without rapid and excessive fatigue [5, 6]. Reduced functional efficiency translates into a deterioration in walking performance and body balance; it also increases the risk of falls [4, 7], which are particularly dangerous for seniors—mortality from falls increases [8, 9].

Gait is a complex biomechanical process that requires continuous brain control [10]. The influence of degenerative changes in the musculoskeletal system on the reduction of gait parameters is beyond doubt. However, studies on the influence of changes in cognitive functions are still in progress. There is a close correlation between gait parameters and dementia development—impaired gait function is an indicator of dementia development in the next 4 years [11]. Cognitive therapies have a positive effect on gait parameters and thus reduce the risk of falls [12].

Knowing that gait parameters and fall risk are influenced by both biomechanical and cognitive processes, it seems reasonable to model the therapy in terms of multitasking (dual-task, DT), where the exercising person simultaneously involves both processes. It has been shown that the difference in the quality of single-task (ST) performance compared to DT is significantly related to cognitive functions [13] and the risk of falls in elderly people [14, 15]. Positive effects of multitasking training involving motor and cognitive functions have been demonstrated in both healthy individuals [16, 17] and those with osteoporosis [18] and Parkinson's disease [19]. At the same time, some studies indicate a lack of improvement in both the physical and cognitive spheres under the influence of DT training [20].

There is a lack of uniform exercise programmes in DT conditions, which, apart from being effective, would be simultaneously available, cheap, and above all safe and attractive for older participants. There is also a lack of standardized guidelines for specific types of additional tasks during DT tests [21]. Such standardization and intensification of DT experiments may bring benefits in the form of a better understanding of the impact of their results on the risk of falling in the elderly [22]. [23] concluded in their systematic review that dynamic exercises (in motion) bring better results than in static conditions (resistance and flexibility), while the simultaneous performance of exercises involving the physical and mental spheres brings better results than performing them separately.

Jaques-Dalcroze Eurhythmics (JDE) is one of still little studied forms of exercises involving both spheres at the same time. To the best of our knowledge, four papers have been published so far describing the impact of JDE on motor skills in older people. Since the results of these studies present JDE as a prospectively effective therapy to improve functional performance in older people, but they are inconclusive at the same time, the authors of this paper decided to study the impact of JDE performance over a 12-week period, twice a week, on the dynamic agility of women over 65 years of age, including multitasking.

## 2. Methods

### 2.1. Participants

The selection of participants was conducted on the basis of advertisements in the local press and on the Internet and covered the area of Poznań agglomeration (Poznań metropolitan area, Greater Poland Voivodeship, West Poland). Qualification criteria included women aged 65+ community-dwelling. Candidates had no contraindications to participate in physical activities and agreed to participate in the experiment. The participants whose results were analysed obtained at least 8 points in the Abbreviated Mental Test Score (AMTS) [24]. Individuals taking medication that disrupts natural control of body equilibrium, using orthopaedic equipment, people with Parkinson's or Alzheimer's disease, people with significant visual and/or auditory perception impairment, and people who undertake or have undertaken regular organized physical activity in the last three years were not qualified for the programme.

**Figure 1:**
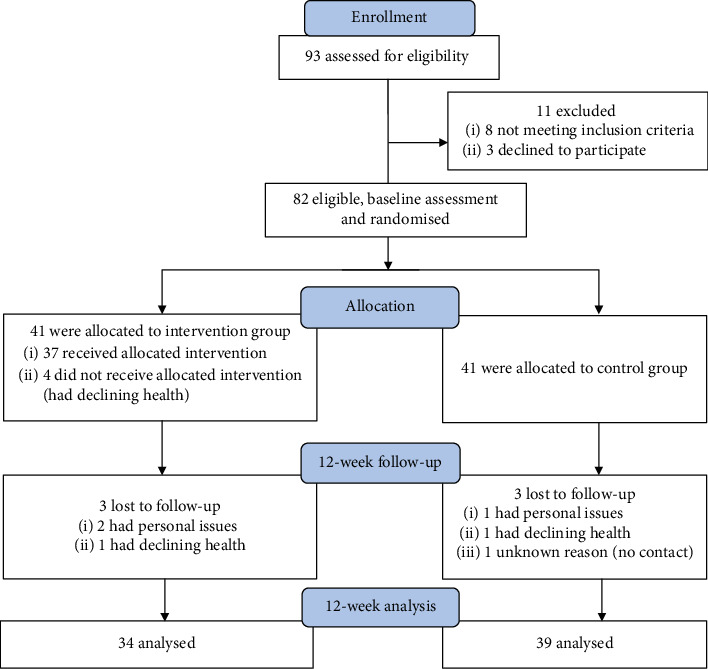
Flowchart for enrollment, randomization, and follow-up of study participants.

As shown in Figure 1, 93 individuals have registered and 82 have qualified for the programme. In the course of randomization carried out using the STATISTICA 10 computer programme (Dell Inc. Tulsa OK., USA), the participants were divided into two equal groups: intervention group (IG) and control group (CG). The final statistical analysis included 73 women (mean ± standard deviation): age 69.9 ± 3.2 years, including 34 with IG (69.7 ± 3.2) and 39 with CG (70.0 ± 3.3). None of the participants had previously experienced JDE, even in theory.

### 2.2. Protocol

The women with IG participated in the physical activity programme, while the CG participants were recommended not to change anything in their existing lifestyle, and in particular not to undertake new, systematized forms of physical activity. The degree of dynamic agility in each participant was assessed twice. The first measurement session (baseline) took place during the week preceding the beginning of exercise and the second one (12-week follow-up) during the week following the end of exercise. The experimenters who made measurements were not aware if participants belonged to the experimental or control group.

The research project was positively assessed by the Bioethics Committee at the Karol Marcinkowski Medical University in Poznań (Resolution 1046/15).

### 2.3. Exercise Programme

Activities were held twice a week for a period of 12 weeks for 45 minutes. Each training session included exercises in the field of rhythmic using the Jaques-Dalcroze Eurhythmics method with piano accompaniment and music played electronically. The activities were conducted by a JDE specialist.

The JDE exercises consisted mainly of recreating the musical course with the use of body movements. Their main idea was to simultaneously engage the motor and cognitive function. Among the exercises conducted were among others movement exercises of rhythmic themes, double and triple speed of movements, double and triple slowing down, rhythmic transformation of themes, and polyrhythms. Apart from rhythmic exercises, inhibition and stimulation of movement, exercises reflecting dynamic, agogic, and articulatory courses in music, improvisation of movements, and exercises shaping independence of movements and their coordination were performed.

### 2.4. Measurements

The Timed Up and Go (TUG) test, described by [25], was used to assess dynamic agility. Each participant received a verbal instruction followed by a show and one training round. The participants then proceeded to the proper tests: first in single-task (TUG_ST) and then in dual-task (TUG_DT) conditions, where the participant counted down loudly from 60 every 3. No explicit instruction for prioritization of either task was given.

The outcome measures consist of TUG_ST, TUG_DT, and the difference between them (dual-task cost, DCT), expressed as a percentage according to the equation: DTC (%) = ((TUG_DT (sec) − TUG_ST (sec))/TUG_ST (sec)) × 100.

### 2.5. Statistical Analysis

All the data were screened and revised regarding missing, skewed, and outlier information. The results of descriptive statistics were presented as averages along with the standard deviation (mean ± SD). The results of TUG tests were shown as averages and confidence intervals of 95%. Intergroup differences were determined using the Mann-Whitney *U* test (with correction for continuity). A repeated two-factor analysis of variance (ANOVA) was carried out. In the case of significant main effects or interactions, Tukey's post hoc test was conducted. The statistical significance level was set at 5%. All calculations were conducted with STATISTICA 10 (Dell Inc. Tulsa, OK).

## 3. Results

**Figure 2:**
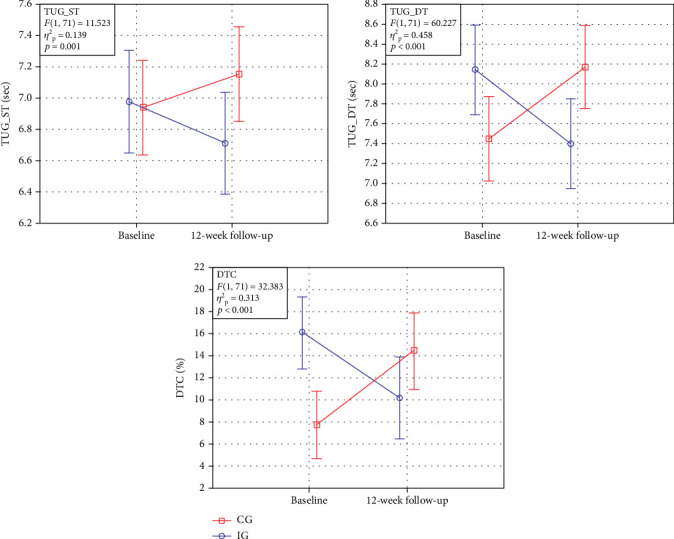
Pre- and postintervention dynamic agility results for Timed Up and Go test in single-task conditions (TUG_ST), Timed Up and Go test in dual-task conditions (TUG_DT), and dual-task cost (DTC).

Table 1 details the results for the two groups in the two periods. The direction of changes in the results obtained by IG and CG before and after the intervention is presented in Figure 2. Significant interaction effects between the group assignment and the measurement session were shown for all measurements (TUG_ST: *F* = 11.523, *η*^2^_P_ = 0.139, *p* = 0.001; TUG_DT: *F* = 60.227, *η*^2^_P_ = 0.458, *p* < 0.001; DTC: *F* = 32.382, *η*^2^_P_ = 0.313, *p* < 0.001). The results of the post hoc test show significant differences in the TUG_DT test between the first and second measurement for IG (*p* < 0.001) and CG (*p* < 0.001). Also in the case of DTC, the significant difference between the first and second measurement for IG (*p* = 0.003) and CG (*p* < 0.001) was found. In the TUG_ST test, post hoc analysis did not show any significant differences (significance of follow-up changes in relation to baseline for IG: *p* = 0.058). No significant main effects were observed.

## 4. Discussion

The influence of JDE exercises on the dynamic agility in seniors was investigated. In each of the three tests (TUG_ST, TUG_DT, and DTC), a significant interaction effect between the assignment to the group and the measurement session was observed: the participation in proposed exercise programme significantly improved the dynamic agility (*p* < 0.001). In CG participants, the results deteriorated—this can be explained by the fact that in older people, especially those with sedentary lifestyles, the ability to control body equilibrium decreases with age [26], similarly to the cognitive functions of the brain [27]. Under ST conditions, none of the groups showed any significant changes between the first and second measurements, while under DT conditions such a difference occurred. It can be assumed that cognitive functions, to a greater extent than physical functions, determine the deterioration of results in DT conditions in CG.

In the aforementioned study [28], people practicing the JDE method for 9 weeks once a week significantly increased their walking speed both in ST and in DT, but in contrast to our results, the DTC index did not change. The improvement of DTC results in our participants may be due to a 3-week longer duration of the programme, its higher intensity, or both. All these possibilities are partly confirmed by World Health Organization data on physical activity of older people at an increased risk of falling, which recommend 3 or more times a week exercise. It seems that the principle “the more the better” works here—even up to 6 times a week for 45 minutes each session of moderate-intensity training is recommended [29]. Therefore, in an easy way, by increasing the frequency of dedicated training programmes, it is possible to improve the effectiveness of proposed therapies.

In the case of the TUG_ST variable, our CG participants did not see any significant improvement in the outcome of their participation in the JDE programme, although at *p* = 0.058, it is possible to refer to a trend. In another study [30], older people improved their performance by about 5% (*p* = 0.02) thanks to their participation in the 6-month JDE test in the TUG_ST test. The difference in results may be due to the difference in the duration of programme—ours was half as long. An additional reason may be a significant difference in the average age of respondents, which translates into significantly different average results obtained in the TUG test. In Trobetti et al., the average age was 75 years and the average TUG_ST test result was 10.25 seconds. In our study, it was, respectively, 70 years and 6.7 seconds.

The aim of this paper was to confirm and enrich the knowledge on the positive influence of the JDE method on the functional efficiency of seniors. Undoubtedly, the strengths of this study are the use of a random controlled trial protocol (RTC), a large research group, and a statistically significant interaction of IG variables with CG in each investigated trait in a relatively short period of time.

The studies carried out are subject to limitations. Above all, it is not possible to determine the changes that occurred in the nervous structures of participants, as no imaging diagnostics of the brain was carried out. Such data could show exactly what is behind the positive changes in IG and negative changes in CG—the proportions between the physical and cognitive spheres could be determined. Furthermore, there were some difficulties with interpreting results, caused by significant differences between results of IG and CG regarding DTC at the baseline, despite properly carried out randomization. Perhaps, increasing the number of participants would level that difference. Additionally, in subsequent studies, it would be advisable to create additional control groups: (1) performing only physical exercises and (2) performing only cognitive exercises. Then, it will be possible to compare and determine more precisely the mechanisms of impact of the JDE method on the dynamic agility of the body.

To sum up, this is the first randomized controlled trial showing a significant effect of JDE exercises, which take place twice a week for 12 weeks and improve dynamic agility control in women over 65 years of age. At the same time, it is worth emphasizing that JDE exercises are attractive for participants, which seem to be a good prognosis for planning and testing effective physical activity programmes for seniors.

## Figures and Tables

**Table 1:** Descriptive data (means and 95% confidence interval (CI)) of the time, in seconds, to perform the Timed Up and Go test without (TUG_ST) and with (TUG_DT) dual-task and the dual-task cost (DTC), in percent, at the baseline and at the 12-week follow-up in both the intervention group and the control group. Significance of differences between groups shown as p-value.

		Baseline		12-week follow-up	
TUG_ST	Intervention	6.98 (6.63-7.33)	*p* = 0.951	6.71 (6.38-7.04)	*p* = 0.039
	Control	6.94 (6.64-7.23)		7.15 (6.84-7.46)	
TUG_DT	Intervention	8.14 (7.57-8.71)	*p* = 0.102	7.40 (6.90-7.91)	*p* = 0.001
	Control	7.45 (7.14-7.76)		8.17 (7.79-8.54)	
DTC	Intervention	16.14 (12.33-19.96)	*p* = 0.001	10.21 (5.86-14.55)	*p* = 0.011
	Control	7.76 (5.14-10.39)		14.43 (11.45-17.40)	

## Data Availability

The datasets analysed during the current study are available from the corresponding author on reasonable request.
